# Understanding liquid–liquid phase separation through TDP-43: fundamental principles, subcellular compartmentalisation, and role of solid inclusion formation

**DOI:** 10.1186/s13059-026-03956-9

**Published:** 2026-01-29

**Authors:** Alessandra Bigi, Fabrizio Chiti

**Affiliations:** Department of Experimental and Clinical Biomedical Sciences, Section of Biochemistry, University of Florence, Florence, 50134 Italy

**Keywords:** Amyotrophic lateral sclerosis, FTLD-TDP, LATE-NC, TAR DNA-binding protein 43, Stress granules, Anisosomes, Paraspeckles

## Abstract

Phase separation is an important process in biology associated with formation of membraneless organelles but possibly related to the emergence of solid inclusions. TDP-43 is a largely studied paradigmatic case, as it forms neuronal cytoplasmic inclusions in neurodegenerative diseases and is an essential component of many membraneless organelles. Here, we review the physicochemical fundamentals of liquid–liquid phase separation (LLPS) of TDP-43 and its fragments in vitro, showing that full-length TDP-43 requires RNA or chaperones to form stable liquid droplets. We describe TDP-43-containing membraneless organelles and the debate on whether these assemblies represent reservoirs for pathological solid inclusion formation.

## Introduction

Liquid–liquid phase separation (LLPS) of proteins is a process in which polypeptide chains pass from a dispersed solution in which they are solvated and adopt their normal native state (either folded or intrinsically disordered) into a second liquid phase enriched with those specific proteins. The resulting mixture is heterogeneous and contains two distinct liquid phases: a classical water solution and the protein-enriched liquid droplets.

LLPS is a remarkable behaviour of proteins that has been known by protein chemists and physicists since the second half of the twentieth century; a great deal of work has been carried out, for example, to characterise the phase diagram of solutions containing bovine γ_II_ crystallins, finding the presence of two distinct liquid phases differing in protein concentration [1, 2]. It was not until 2009, however, that LLPS of proteins was found to be a relevant phenomenon in cells to achieve a functional compartmentalisation of RNAs and proteins in the absence of lipid bilayers and, therefore, departing from the classical view of membrane-containing organelles [3]. Indeed, in 2009 it was reported for the first time that the one-cell embryo of *C. elegans* has P granules containing RNAs and proteins that exhibit liquid-like characteristics, including coalescence, dripping and wetting, allowing typical characteristics of the liquid state of matter to be measured, such as viscosity and surface tension [3]. Two years later, the LLPS properties of the nucleolus, which represents the largest membrane-devoid organelle in eukaryotic cells known since the end of the nineteenth century, were described in oocytes from *Xenopus laevis* [4]. Shortly thereafter, it was reported that interactions among multivalent macromolecules, including proteins and RNAs, lead to LLPS and generate µm-sized liquid droplets; this phenomenon was proposed to be a cardinal strategy to spatially organise and biochemically regulate information in the cell [5].

A large number of proteins and RNAs have today been converted in vitro into liquid droplets through LLPS. The number of proteinaceous and RNA-containing liquid droplets described in cell biology and forming through LLPS has also continued to grow since 2009, leading to the term of “membraneless organelles” (MLOs) to describe them [6, 7]. This term remarks the absence, on the one hand, of a surrounding lipid membrane, unlike classical organelles, and also underlines, on the other hand, the compartmentalised nature typical of cell organelles. MLOs have the ability to exchange molecules with the surrounding environment and show a high degree of spatiotemporal coordination of biochemical reactions. Hence, LLPS plays a crucial role in a variety of physiological processes, but it may also play a key role in the development of pathogenic conditions, such as cancer and neurodegenerative disorders [8–10]. A list of the most highly studied proteins undergoing LLPS in vitro and in cells is included in Table 1.

**Table 1:** List of most highly studied proteins undergoing LLPS, with evidence both in vitro (purified protein) and *in cellulo* (as MLOs)^1^

**Protein**^**1**^	**Evidence of LLPS in vitro (purified protein)**	**Evidence of LLPS in biology (protein forming MLOs)**
Fused in sarcoma (FUS)	Pure and full-length [11]	Stress granules [12] P-bodies [13]
TAR DNA-binding protein 43 (TDP-43)	Only with co-factors [14], or fragments [15, 16]	Stress granules [17], SG-independent droplets [18], P-bodies [19], transport granules [20], paraspeckles [21], Cajal bodies/gems [22], promyelocytic leukemia nuclear bodies [23], nuclear stress bodies [24], anisosomes [25]
Tau (τ)	Pure 4R-protein K18 [26]	Tau droplets to grow MT [27] stress granules [28]
α-synuclein (αS)	Pure and full-length [29]	α-synuclein droplets [29] P-bodies [30]
ATP-dependent RNA helicase laf-1 (LAF-1)	Pure and full-length and fragments [31, 32]	P granules [31]
Heterogeneous nuclear ribonucleoprotein A1 (hnRNPA1)	Pure and full-length [33]	Stress granules [33]
Bromodomain-containing protein 4 (Brd4)	Pure and full-length [34]	Nuclear puncta [35]
Heterochromatin Protein 1α (HP1α)	Phosphorylated mutants [36–38]	Nuclear puncta [38]
Tia1 cytotoxic granule associated RNA binding protein (Tia1)	Pure and full-length, disease-associated mutants [39, 40]	Stress granules [39, 40]
Annexin A11 (A11)	Pure and full-length [41]	RNA granules [41]
DEAD-Box Helicase 4 (DDX4)	Pure but only N-terminal domain [42]	Various MLOs [43]
TATA-binding protein-associated factor 2 N (Taf15)	Pure and full-length [44]	Nuclear puncta at sites of DNA damage [45]
Nephrin	Engineered proteins [5]	Micron-scale domains at the basal plasma membrane [46]

^1^The table includes all proteins with evidence of formation of droplets through LLPS, both in vitro as a purified protein and in cells as MLOs with other factors. The table is not comprehensive but includes the proteins for which LLPS behaviour is most highly studied according to *Pubmed* database. For a comprehensive list see PhaSePro (https://phasepro.elte.hu/browser)

The three hallmarks of liquid protein condensates formed in vitro from purified systems through LLPS are today considered to be (i) a roughly spherical shape adopted due to surface tension, (ii) an ability to coalesce and (iii) an ability to undergo rapid internal diffusion and molecular rearrangement [47]. These are also used as gold-standard criteria to define protein assemblies as liquid droplets [47]. In cells, they cannot be adopted in their entirety to identify liquid protein droplets, mainly due to difficulties to detect their round morphology and ability to coalesce. For this reason, a cellular structure needs to fulfill other related requirements to be considered an MLO: (i) to be microscopically visible as an entity not surrounded by a lipid membrane, (ii) to be enriched with specific factors typical of that organelle, (iii) to be often continuously exchanging with the surrounding environment [48].

### TDP-43 as a paradigmatic protein associated with neurodegeneration and undergoing LLPS

A growing body of evidence indicates that many of the pathogenic proteins associated with neurodegenerative diseases phase-separate under both physiological and pathological conditions [26, 29, 49–52]. In this review, we will describe TAR DNA-binding protein 43 (TDP-43) as an example of the proteins undergoing LLPS, as it is clearly one of the paradigmatic cases for studies of LLPS of proteins in vitro and *in cellulo*. Indeed, it is associated with a variety of clinically diverse neurodegenerative diseases, it is involved in many different MLOs in biology and a wealth of information is present in the literature that clarify generic concepts and help understand other systems.

TDP-43 was first discovered in 1995 for its role in binding the TAR DNA of the HIV virus [53] and was later recognised to be a DNA- and RNA-binding protein involved in gene transcription, mRNA splicing, stabilisation, maturation and translation [54–56], with over 6000 RNA species known to be TDP-43 binders nowadays [57]. In 2006, the cytoplasmic neuronal protein inclusions that had remained elusive until then and that are associated with non-familial amyotrophic lateral sclerosis (ALS) and ubiquitin-positive, tau-negative frontotemporal lobar degeneration (FTLD-U), were found to consist of TDP-43 [58, 59]. Today we know that TDP-43 solid inclusions are found in all cases of sporadic ALS (90%), most cases of familial ALS (around 60–70%), in 50% of cases of FTLD (renamed FTLD-TDP), as well as limbic-predominant age-related TDP-43 encephalopathy (LATE), which mimics Alzheimer's-type dementia, and Perry disease (PeD) [60–63]. TDP-43 inclusions are also detected in many other neurodegenerative diseases, but in all these cases they are not thought to be the primary neuronal lesions.

Under such pathological conditions TDP-43 mislocalises from the nucleus to the cytosol, where it forms solid inclusions, readily detectable by histological analysis of *postmortem* patients [58–63]. Experimental evidence collected in animal and cell culture models suggests that TDP-43 proteinopathies arise from both a loss-of-function, due to nuclear depletion of functional TDP-43, and a gain-of-function due to accumulation of aberrant TDP-43 inclusions [64–67]. Post-translational modifications (PTMs) are observed in the inclusions, including polyubiquitination, hyperphosphorylation, fragmentation [58–60], acetylation [68], SUMOylation [69], and also citrullination and monomethylation in type A FTLD-TDP [70]. Hyperphosphorylation is a key PTM, defining the so-called TDP-43 pathology [58, 59].

Under conventional immunohistochemistry, the inclusions appear either diffuse and granular, compact and round, or filamentous and skein-like [71–73]. Diffuse/granular inclusions are small (< 1 µm), lack a defined morphology and are numerous within individual neurons [71–73]. Round inclusions are 1–25 µm, present as 1–2 units per neuron and approximately spherical and compact [60, 71–74]. Skein-like inclusions are *ca*. 0.5–1.0 µm in diameter and up to 15 µm in length [60, 71–74]. Under transmission electron microscopy (TEM), 10–20 nm wide filaments containing TDP-43 are observed, particularly in skein-like inclusions; this fibrillar texture is still evident in round inclusions but absent in diffuse/granular assemblies [73–80].

Using cryo-electron microscopy (cryo-EM), the structures of the protease-resistant portion of filaments extracted from patients have been solved to a 2.6 Å resolution [70, 81]. In both solved folds, the β-sheets did not stack on each other in the typical cross-β structure of amyloid fibrils that provides the 10–11 Å reflection in X-ray fibre diffraction [70, 81] and do not present deep and non-polar grooves to bind amyloid-diagnostic dyes [81], explaining why dye binding remains often undetected in pathological TDP-43 inclusions [82–85]. Accordingly, amyloid-like structural and tinctorial properties were not found in TDP-43 inclusions obtained with heterologous overexpression in bacteria [86], eukaryotic cells [87], or after inducing aggregation of purified TDP-43 [87, 88].

Solid-phase inclusions are not, however, the only type of self-assemblies that many neurodegeneration-associated proteins form, including TDP-43. It is now well established that TDP-43 participates to the formation in cells, under physiological conditions, of droplets in both the nucleus and cytosol (MLOs), in association with other proteins and RNA molecules. In MLOs TDP-43 forms both homotypic and heterotypic interactions [57]. MLOs are very important in cell biology to compartmentalise specific molecules and functions and are also studied as possible initiators of solid inclusion formation. This has paved the way to a wide range of studies of TDP-43 LLPS, both in vitro in a non-cellular context to reveal its fundamental physicochemical principles, and in cells to study its relevant biological and pathological implications, to an extent that is unprecedented.

In this review, we will start by summarising the structure of native TDP-43 and will then review results on phase separation of pure TDP-43 and its fragments in vitro, with the aim of disclosing the physicochemical fundamentals governing this process. We will finally describe the various cytoplasmic and nuclear MLOs containing TDP-43 that have been described so far, particularly those that are either more informative or studied in more detail, with an emphasis on the existing debate on whether they represent nuclei for the formation of pathological solid inclusions.

### Structure and functional oligomerization of native TDP-43

**Fig. 1:**
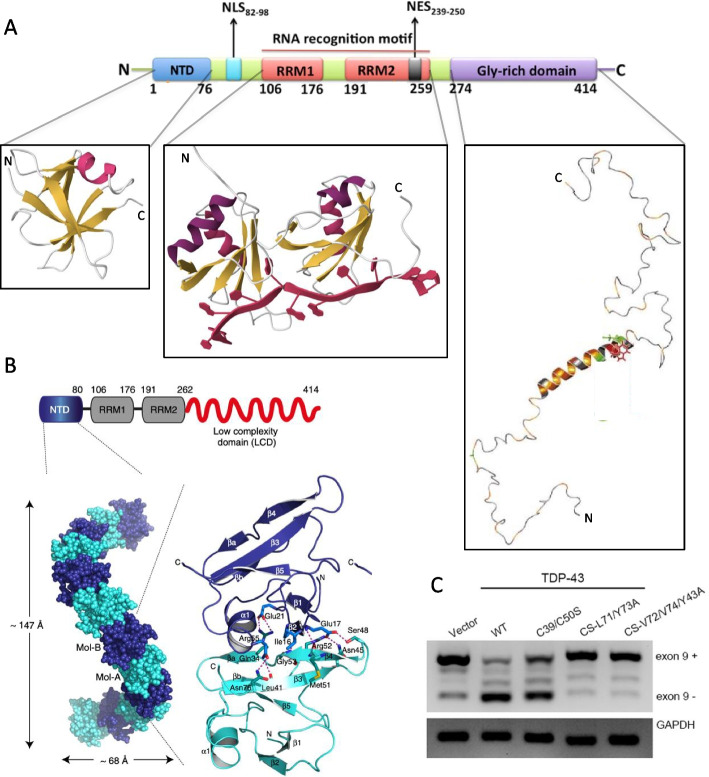
Structure and oligomeric structure of native TDP-43. **A** Domain composition of the TDP-43 sequence (top) and ribbon diagram structures of its four domains (bottom). The DNA molecule bound to RRM1/RRM2 is indicated in red. Domain composition on top is reproduced from [100]. Crystal structure of NTD and NMR solution structure of RRM1/RRM2 are from PDB entries 5MDI [91] and 4BS2 [93], respectively. The CTD structural model is adapted from [98]. **B** Whole atom crystal structure of TDP-43 NTD with single NTD molecules arranged in head-to-tail fashion. Blue and cyan colours indicate alternating NTD molecules. The ribbon structure on the right indicates the monomer–monomer interface. Reproduced from [91]. **C** Suppression of TDP-43 function upon NTD oligomer destabilization in HEK293T cells. Lanes indicate transfection with control vector without CFTR exon 9 exclusion (lane 1), with vector overexpressing WT TDP-43 promoting CFTR exon 9 exclusion (lane 2), TDP-43 with C39S/C50S double mutation with partial decrease in CFTR exon 9 exclusion (lane 3), TDP-43 with C39S/C50S double mutation plus dimer interface destabilizing L71A/Y73A and V72A/V74A/Y43A mutations with abolishment in CFTR exon 9 exclusion (lanes 4–5). Reproduced from [90]

TDP-43 is a ubiquitous nuclear protein containing 414 amino acid residues and consisting of a folded N-terminal domain (NTD_1–76_), belonging to the DIX domain structural family according to SCOP 2 [89–92], two folded RNA recognition motifs (RRM1_106–176_ and RRM2_191–259_) [93–95], and an intrinsically disordered C-terminal domain (CTD_274–414_), also called low-complexity domain (LCD), prion-like domain (PrLD) or glycine-rich domain (GRD), which is intrinsically disordered with an amphipathic α-helix spanning approximately amino acid residues 320–340 [15, 96–98] (Fig. 1A). TDP-43 also contains a nuclear localisation signal (NLS_82–98_) and possibly a nuclear export signal (NES_239–250_), which allow it to shuttle between the nucleus and the cytoplasm (Fig. 1A) [99].

TDP-43 was initially proposed to form a dimer through interactions involving NTDs between adjacent molecules [100–105]. Later on, isolated NTDs, and consequently full-length TDP-43 molecules via NTDs, were found to form head-to-tail interactions between two distinct NTD surfaces, leading to a propagation of the dimeric state to form an oligomer of undefined length (Fig. 1B) [91, 92, 106]. Unlike monomeric TDP-43, the dimeric/oligomeric state is functional, as mutants of full-length TDP-43 having deletions or substitutions of residues necessary for NTD oligomerization lose their ability to regulate TDP-43 mediated splicing of target mRNAs, while still allowing protein folding (Fig. 1C) [89–91].

### The PrLD of TDP-43 undergoes genuine LLPS in vitro

**Fig. 2:**
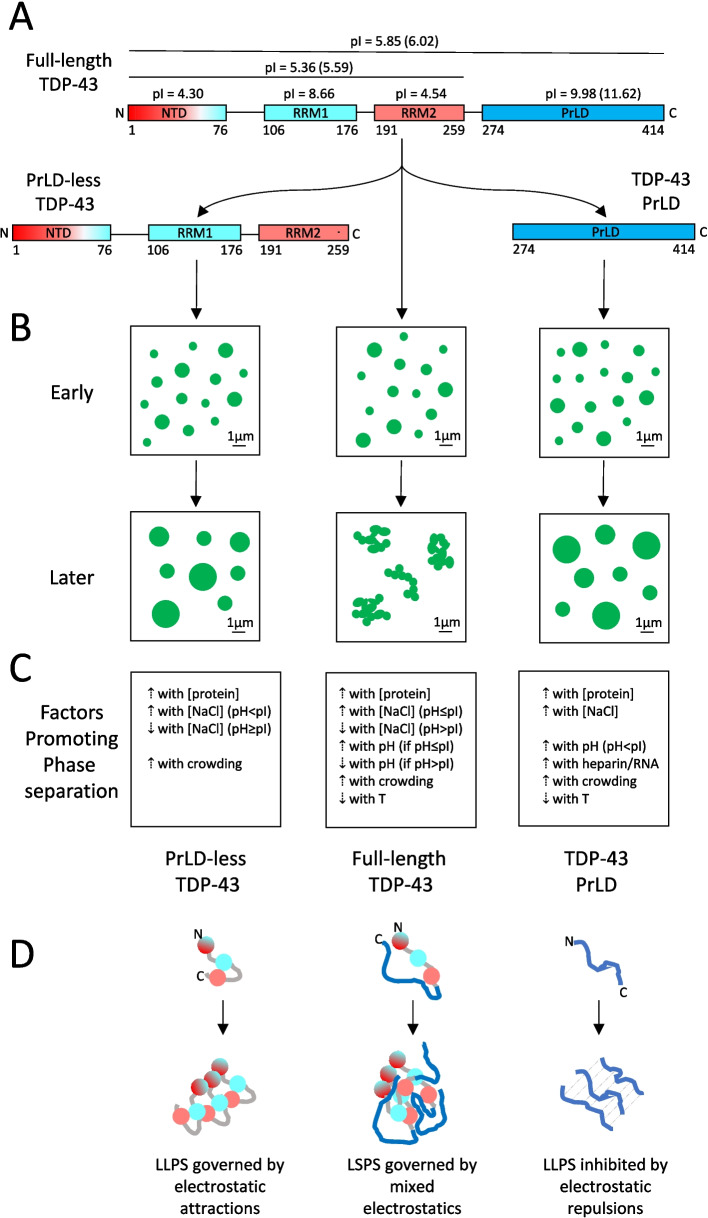
Electrostatic forces governing phase separation of PrLD-less, PrLD and full-length TDP-43. **A** Domains of full-length (centre), PrLD-less (left) and PrLD (right) TDP-43. Domain net charges at neutral pH are negative (red), weakly positive (pale blue), or positive (blue). pIs are indicated (pIs with a 6 His-tag in brackets). **B** Phase separation types observed early (top) and later (bottom) for the indicated TDP-43 constructs. Only PrLD-less (left) and PrLD (right) fragments form persistent liquid droplets. Full-length TDP-43 forms gel-like droplets (middle). **C** Factors governing phase separation for the three species. **D** Scheme of electrostatic forces modulating phase separation for the three constructs (colours as in A). Adapted with permission from [110]

Due to problems of poor solubility, fragmentation and aggregation of full-length TDP-43, the first studies of phase separation in vitro were carried out with the PrLD [15, 50, 97, 98, 107, 108], which was also thought to be the most important portion of TDP-43 due to its low complexity sequence, presence of C-terminal fragments in pathological inclusions and locus of over 90% of ALS-associated mutations (reviewed in [109]). A seminal paper from Fawzi’s group showed that the isolated TDP-43 PrLD is able to form, under appropriate conditions, round assemblies undergoing coalescence and internal molecular diffusion, as monitored with FRAP, therefore recapitulating all three hallmarks of LLPS [15], as simplified in our scheme (Fig. 2A, B).

This observation was later confirmed in many different laboratories. Today we know that LLPS of TDP-43 PrLD is promoted by increases of PrLD concentration [15, 50, 98, 111], pH [50, 112, 113], salt concentrations [15, 50, 98, 111, 113], addition of negative polyelectrolytes such as ATP, DNA, RNA and heparin [15, 108, 112–114], neutral crowding agents [50], chemical chaperones like trimethylamine N-oxide [107] and low temperatures [50, 98], whereas small concentrations of urea and 1,6-hexanediol (HD) inhibit the process [98]. The positive effects of high protein concentrations, low temperatures and neutral crowding agents can be explained by the physical forces governing generic phase separation [115]. By contrast, the precise dependences on pH, salts, ATP and negative polyelectrolytes, as well as urea/HD, have led to the conclusions that PrLD LLPS is inhibited by electrostatic repulsions and promoted by hydrophobic interactions [50, 98, 111–114], particularly by interactions between aromatic residues, as reported later [116, 117]. With these forces governing the process, a pH increase promotes LLPS because the PrLD isoionic point (pI) is very high (9.98 or even 11.62 when a His-tag is present) and a pH increase attenuates repulsion between the positively charged residues at the N-terminus of the PrLD, bringing the protein domain closer to neutrality. Similarly, salts/ATP/heparin/RNA promote LLPS because they shield repulsions, which is particularly effective given the polymeric nature of the two latter agents.

More precisely, ATP/RNA/heparin have an LLPS-promoting effect only at small concentrations, as higher concentrations disrupt LLPS through a “reentrant” behaviour [70, 112–114]. It was hypothesised that initial addition of these agents resulted in PrLD charge neutralisation with consequent LLPS, whereas their subsequent addition causes an exacerbation of the negative charge in the overall polyelectrolyte/protein system with a hydrotrope effect, resulting in electrostatic repulsion and consequent droplet disruption [113, 114]. In addition, using both experimental and simulation approaches on LLPS of wild-type PrLD and phosphomimetic mutants (S403D/S404D/S409D/S410D), it was concluded that ordinary salts promote wild-type PrLD LLPS by screening electrostatic repulsions, but also by increasing the hydrophobic effect due to their kosmotropic nature [111]. However, at high salt concentrations (above approximately 250–500 mM), the positive charges are saturated, and the screening effect is no longer present, with salts still promoting LLPS to a lower extent following only the hydrophobic effect [111].

More recently, the importance of α-helix_320–340_ in PrLD LLPS, which is the only structured element of the domain and whose role had been recognised earlier [15, 50, 96–98], was studied in more detail [114, 118, 119]. Mutations that stabilise and destabilise (or delete altogether) the helical structure in this region were found to increase and decrease dramatically LLPS of the entire PrLD, respectively [114, 118, 119]. It was also reported that in dynamic PrLD assemblies, interactions between hydrophobic residues of adjacent α-helices_320–340_, unlike hydrophilic ones, mediate the process; in particular, a methionine-rich core plays a relevant role in this dynamic core with other contributions from tryptophan and leucine residues [120].

### PrLD-less TDP-43 also undergoes genuine LLPS in vitro

Two recent reports have shown that the complementary fragment of the TDP-43 PrLD containing the first three folded domains but lacking the PrLD (generally called PrLD-less TDP-43), is also able to phase separate into liquid droplets in vitro under a variety of conditions [16, 110], as simplified in our scheme (Fig. 2A, B). In both reports, PrLD-less TDP-43 labelled with a fluorescent probe was found to form round droplets, with an ability to coalesce and recover its relative fluorescence intensity (*RFI*) after photobleaching using FRAP [16, 110]. A shorter fragment containing the two RRM domains was still able to phase separate, albeit with much lower efficiency and only in some conditions, whereas the NTD alone was not, indicating that the two RRM domains are the main mediators of the process, with the NTD having a promoting effect [16].

At pH values close or higher than the pI (5.36 or 5.59 with a His-tag), LLPS of PrLD-less TDP-43 is inhibited by salts, unlike the complementary PrLD fragment (Fig. 2C) [16, 110]. This indicates that demixing of this protein construct is promoted by electrostatic attractions, which can be explained by considering the importance of the two RRM domains in the process and that they have pIs of 8.66 and 4.54, respectively (Fig. 2A, D), and, therefore, opposite net charges at both the pH of 5.5 and 7.0 studied [110]. By contrast, at pH 4.0 all three domains of PrLD-less TDP-43 are positively charged (Fig. 2A) and LLPS is promoted by NaCl, rather than inhibited, because it acts as a shielding factor of the electrostatic repulsions between PrLD-less molecules, in analogy with the positively charged PrLD at all pH values [110].

### Full-length TDP-43 undergoes unclear LLPS in vitro

Unlike PrLD and PrLD-less TDP-43, it was repeatedly reported that in absence of other cofactors pure full-length TDP-43 does not retain a clear LLPS ability in the test tube, or that at least it forms round droplets that convert very rapidly into gel-like species (see references below). In fact, it has been found by many investigators and under different conditions, that full-length TDP-43 self-assembles rapidly into spherical species unable to coalesce into larger droplets and exhibiting weak or no recovery of *RFI* after photobleaching (Fig. 2A, B).

Using confocal fluorescence microscopy, for example, it was shown that TDP-43 fused to the yellow fluorescent protein (YFP-TDP-43) forms speckles that assembled further, without fusing, into irregular flocculent tufts featuring partial FRAP [83]. Initially round assemblies of TDP-43 were later unable to fuse and rather accumulated in strings or formed irregularly shaped aggregates [33, 92]. Partial cleavage of TDP-43 fused to the maltose binding protein (TDP-43-MBP) to release untagged TDP-43 led to the formation of condensates that assembled in a chain-like arrangement and then clustered further, without fusion and with only partial FRAP [121]. In the absence of any solubilizing large tags, when diluted from its initial solution that kept it soluble, TDP-43 self-assembled rapidly into a number of solutions, specifically at 2.5–10 µM protein, pH 4–7, 0–150 mM NaCl, with or without 5% (w/v) PEG8000 or TCEP, 20–43 °C [122]. The assemblies were round in shape, but they were unable to fuse and did not show any internal molecular diffusion with FRAP [122]. Similarly, among six studied proteins, TDP-43 fused to Enhanced Green Fluorescent Protein (TDP-43-EGFP) was the one with the highest difficulty to form round and fusing assemblies [34]. To our knowledge, there is only one report on SUMO-TDP-43 and TDP-43-MBP fused proteins in which the droplets appeared spherical and able to fuse, therefore showing LLPS [123]. Hence, it appears that TDP-43 forms liquid round droplets, that are, however, thermodynamically and kinetically unstable and convert rapidly into gel-like and solid species unable to coalesce.

TDP-43 phase separation has a clear pH dependence, with a peak at pH 6.0, which is close to its pI of 5.85 (or 6.02 with the His-tag), and then a decreased efficiency as the pH departs from the pI in both directions (Fig. 2C) [122]. Salt promotes and inhibits this process up to the pI and above, respectively (Fig. 2C) [122]. These precise relationships suggest that neither the PrLD nor the PrLD-less fragment mediate TDP-43 phase separation by themselves, without the contribution of the other domains, as we should expect their own individual pH and salt dependences, which is not the case. It rather appears that full-length TDP-43 self-assembles through the cooperation of all its domains (Fig. 2D), as a whole protein system through both electrostatic and hydrophobic interactions, which seem the dominant forces in phase separation more generally [124, 125]. In this complex process, which is maximised at the pI of the full-length protein rather than those of individual or grouped domains, salts have a positive or negative effect, depending on whether electrostatic repulsions or attractions are dominant. The multiple and perhaps sufficiently strong interactions between heterogenous domains break the multivalent and low-affinity contacts that are necessary for LLPS, allowing the rapid conversion into a gel-like/solid phase (Fig. 2D).

### Genuine LLPS of full-length TDP-43 is facilitated by RNA and protein cofactors in vitro

*Bona fide* and kinetically stable liquid droplets easily form if RNA or DNA oligonucleotides are added to purified full-length TDP-43 [14, 126, 127] or to cells extracts after TDP-43 expression [14]. LLPS of TDP-43 PrLD is also facilitated by DNA, RNA or other physiological polyelectrolytes [15, 108, 112, 113] and small concentrations of single-stranded DNA or poly(ADP-ribose) also make it possible NTD_1–102_ (but not NTD_1–80_) and RRM1-RRM2_97–261_ LLPS [108, 123, 128]. Moreover, chaperone-containing, RNA-free, liquid droplets of TDP-43 have been found in cell cultures [25, 129], although evidence of reconstituted TDP-43/chaperone droplets in vitro is still awaited. Stable liquid droplets can also form in vitro by adding TDP-43 in the presence of RNA and G3BP1 in a stress granule (SG) reconstitution system [130]. These results indicate that physiological factors such as RNA and chaperones may stabilise the liquid properties of TDP-43 assemblies, providing an explanation as to why LLPS of TDP-43 is frequently observed in cells and why a great deal of TDP-43-containing MLOs exist physiologically, as described in the following sections.

### TDP-43 is involved in a large variety of cytoplasmic and nuclear MLOs

**Fig. 3:**
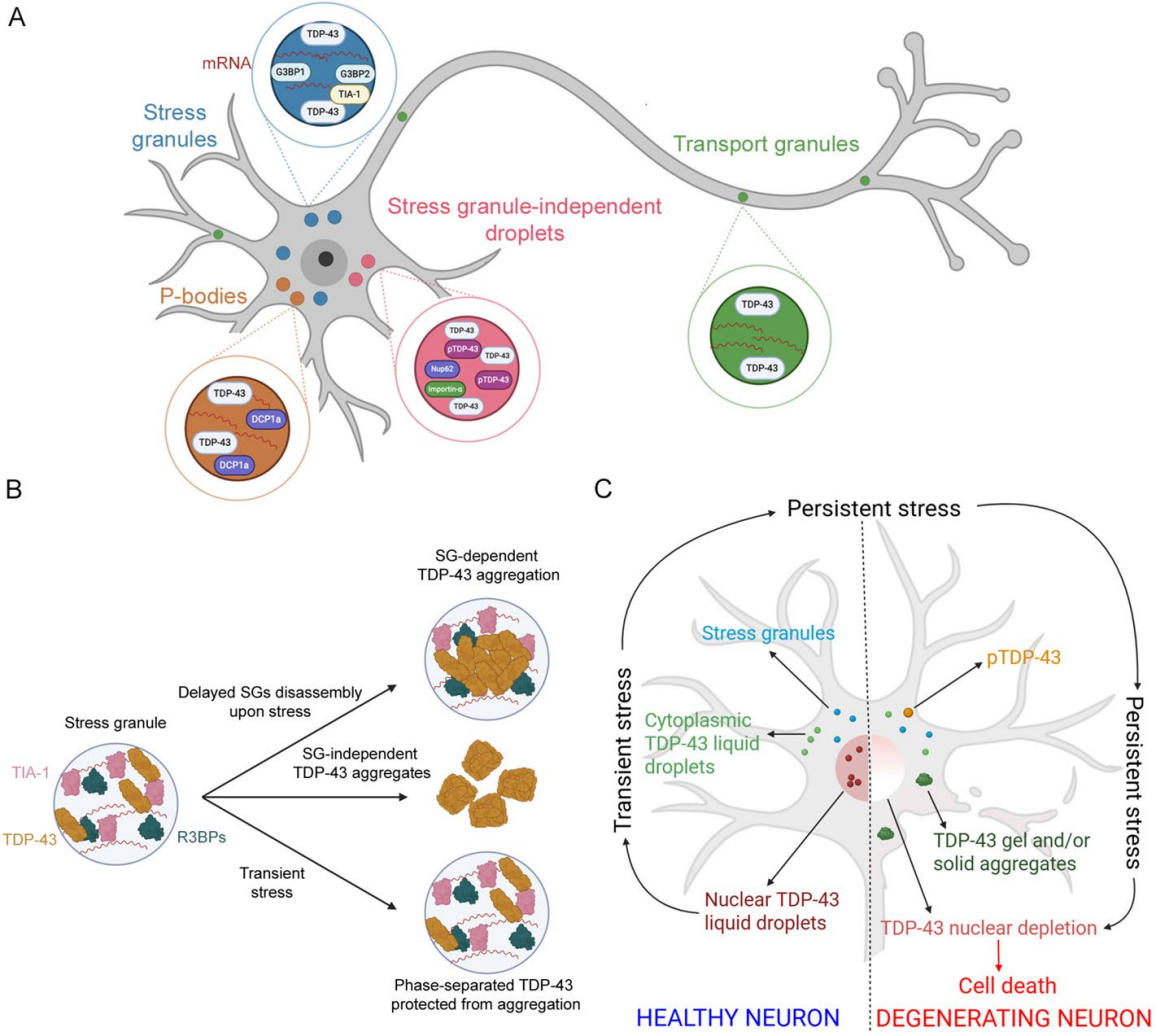
TDP-43 LLPS in the cytoplasm. **A** An overview of the main cytoplasmic MLOs in which TDP-43 is recruited. **B** The hypothesised roles of SGs in TDP-43 cytoplasmic aggregation as detrimental (top), neutral (middle) or protective (bottom). **C** Transient stress induces cytoplasmic SGs and TDP-43 demixing independent of SGs, leading to the formation of long-lived droplets inducing nuclear import defects, nuclear TDP-43 clearance, solid inclusions, and cell death. Adapted and redrawn from [18]. Created with BioRender.com

**Fig. 4:**
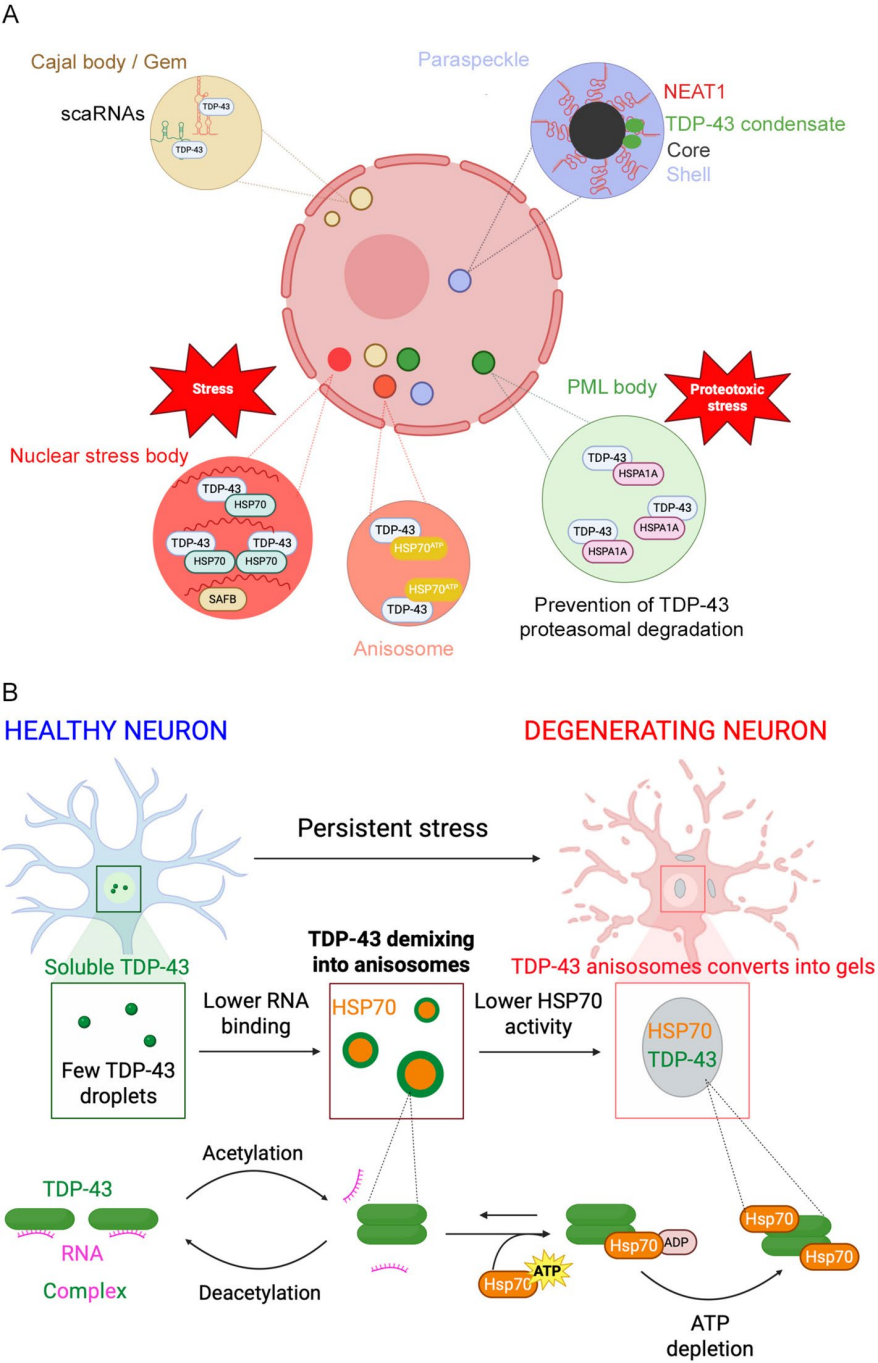
TDP-43-containing condensates in the nucleus. **A** An overview of the main nuclear condensates in which TDP-43 is recruited. **B** Formation of nuclear anisosomes in degenerating neurons in which TDP-43 loses its capability to bind RNA in the presence of disease-associated mutations or post-translational acetylation. Adapted and redrawn from [25]. Created with BioRender.com

A number of MLOs of both nucleus and cytoplasm have been described to accumulate TDP-43 either as a result of stress or specific functions, making this protein as one of the protein systems more represented than anyone else in such organelles, and emphasising the importance of its study in this context as a paradigmatic case. TDP-43 containing MLOs are, in the cytoplasm, stress granules [17], stress-granule independent droplets [18], P-bodies [19], and neuronal transport granules [20] and, in the nucleus, paraspeckles [21], Cajal bodies and gems [22], promyelocytic leukemia (PML) nuclear bodies [23], nuclear stress bodies (NSBs) [24] and anisosomes [25]. All of them are depicted schematically in Figs. 3A and 4A. In the following sections, we describe the most relevant cytoplasmic and nuclear MLOs to which TDP-43 is recruited and discuss their possible roles in pathogenesis.

### Cytoplasmic stress granules: a well-studied example of MLOs that recruit TDP-43

SGs represent the most classical and known example of MLOs forming through phase separation (Fig. 3A). They are reversible and dynamic non-membranous protein/RNA complexes triggered by an array of stressors, to protect RNA from degradation and hamper translation initiation, promptly disassembling upon stress removal and returning mRNAs for translation [131–133]. They have also been proposed to be part of the proteostasis network to handle aggregation-prone proteins [49]. TDP-43 represents a well-studied protein partitioning into SGs, since its identification in these MLOs upon exposure of motoneuron-like NSC-34 cells to oxidative stress, proteasome inhibition and heat shock [17]. A specific sequence segment comprising residues 216–315 and involving parts of the RRM2_191–259_ and CTD_274–414_, together with the RRM1_106–176_, are primarily implicated in TDP-43 recruitment at SGs [17]. Following evidence confirmed the presence of TDP-43 in SGs and revealed its role in their assembly, maintenance and disassembly by modulating the expression of specific RNA-binding proteins such as Ras GTPase-activating protein-binding proteins 1/2 (G3BP1/2) and T-cell intracellular antigen-1 (TIA1), in both transformed and primary cell lines [134–137], as well as in an in vitro model of primary neuron ageing in which decreased TDP-43 protein levels had a negative impact on SG dynamics [138].

### SGs in TDP-43 pathology: detrimental, neutral or even protective?

It is not yet clear if SGs mediate, are irrelevant, or even prevent the formation of TDP-43 inclusions. The previously described enrichment of TDP-43 in SGs has led to the hypothesis that these MLOs may act as “crucibles” of cytoplasmic TDP-43 aggregation. Consistently, colocalisation between TDP-43 inclusions and SGs markers has been detected in both pathological brain tissues and cultured human BE-M17 neuroblastoma cells, suggesting that they could derive from degenerated SGs [73, 134, 139] through the transition from liquid to solid entities potentially able to seed the formation of TDP-43 inclusions [140]. In the same line, the depletion of ataxin-2, a cardinal SG component, has been reported to alleviate TDP-43 neurotoxicity in a rat model of inherited ALS [141]. The delay of SG disassembly during recovery upon stress has been associated with the formation of TDP-43 cytoplasmic aggregates, leading to the exacerbation of TDP-43 pathology and neurodegeneration in *Drosophila melanogaster* [142]. Very recently, Hyman and coworkers revealed that oxidative stress and the abnormally high concentration of TDP-43 within SGs cause SG-dependent TDP-43 aggregation in HeLa cultured cells and iPSC-derived motoneurons [130]. When concomitant, these conditions induce intra-condensate demixing, with the formation of a dynamic TDP-43 enriched phase within SGs, which subsequently transitions into pathological aggregates [130] (Fig. 3B, upper panel). Similarly, it has been demonstrated that aggregation of fused in sarcoma (FUS) protein also initiates within SGs, with ALS-associated mutations promoting FUS accumulation in SGs, and FUS aggregates colocalising with SG markers in ALS/FTD *postmortem* brains [143, 144].

Other studies suggest that solid TDP-43 inclusions can form either within or independently of SGs. In particular, Mann and collaborators used human embryonic kidney HEK293 cells to demonstrate that the formation of neurotoxic TDP-43 aggregates is independent of SGs and rather induced by the aberrant interactions between CTD_274–414_ domains outside SGs [145]. Similarly, human embryonic kidney QBI-293 cells exposed to arsenite or expressing aggregation-prone ∆NLS TDP-43, as well as *postmortem* ALS motoneurons, were found to contain TDP-43 inclusions distant from SGs [146]. Chronic oxidative stress applied to human fibroblasts and iPSC-derived motoneurons from both healthy and ALS individuals induced the formation of granular inclusions containing phosphorylated TDP-43 that generally did not colocalise with SGs [147]. Consistently, it was recently shown that the formation of large, solid-like and neurotoxic TDP-43 inclusion can occur within SGs or outside them in NSC-34 cells overexpressing TDP-43 [148]. Along the same lines, Streit and coworkers took advantage of human H4 neuroglioma cells under stress to show that TDP-43 can aggregate in the cytoplasm independently of SGs, because the protein undergoes a global, reversible decrease of mobility in all cellular compartments under stress, independently of its localisation within SGs [149]. Studies in both yeast and human cell lines demonstrated that the formation of cytoplasmic TDP-43 inclusions can be initially facilitated by the association with SGs, that are, however, not essential to this process, as indicated by the absence of a direct relationship between the ability of the cells to form SGs and the amount, aggregation and cytotoxicity of TDP-43 [150, 151] (Fig. 3B, middle panel).

On the other hand, a potentially protective role for SG localisation of TDP-43 was hypothesised in COS-7 kidney fibroblast-like cells, where proteinaceous foci excluded from SGs rapidly transitioned into disease-associated and hyperphosphorylated inclusions [123] (Fig. 3B, lower panel). Phosphorylation and aggregation of the pathologically relevant C-terminal fragments C35/C25 were proposed to arise from their inability to phase separate and colocalise with SGs [123]. Interestingly, Choi and coworkers took advantage of the chemical chaperone trimethylamine N-oxide (TMAO) to decouple in vitro liquid condensation of pure TDP-43 PrLD from its pathological solid aggregation, suggesting that selective targeting of the latter is a valuable strategy to prevent aggregation [107].

Notably, the same systematic study by Hyman and co-workers described above also reconciled convincingly all these different views across cultured cells, iPSC-derived motoneurons, mouse models and patient samples from both ALS and FTLD [130]. While TDP-43 recruited to SGs is not yet pathological, it is a demixing event, in which liquid puncta enriched with TDP-43 appear at the periphery of SGs, that promotes its solid aggregation. Liquid demixed puncta rapidly convert into solid aggregates under conditions of oxidative stress, driven by oxidation of cysteine residues, particularly of the locally unfolded RRM1 domain, and interactions and α-to-β transitions of α-helices_320–340_ [130]. TDP-43 LLPS/aggregation was also shown to occur independently of SGs, but only upon TDP-43 overexpression, when its global or local cytoplasmic concentrations exceed a critical threshold [130].

### TDP-43 beyond SGs: cytoplasmic demixing and myo-granule assembly

SGs are not the only MLOs formed in the cytoplasm under stress conditions. In human cell lines, including iPSC-derived neurons, transient stress induced by arsenite exposure promotes TDP-43 demixing into cytoplasmic liquid droplets independent of SGs, that rapidly convert into solid-like inclusions recruiting phospho-TDP-43 (Fig. 3A) [18, 149]. Moreover, an increased concentration of TDP-43 in the cytoplasm, or the transient exposure to fragmented amyloid-like fibrils, evoked the formation of persistent, long-lived SG-independent droplets populated by phospho-TDP-43 [18]. This suggested that such liquid species may mature to a gel-like solid state that ultimately nucleates TDP-43 aggregation (Fig. 3C) [18]. These droplets were also able to recruit importin-α and components of the nuclear pore such as Nup62 protein and induce the mislocalization of the RanGap1 protein, normally implicated in TDP-43 nuclear import, thus slowly depleting nuclear TDP-43 and ultimately eliciting cell death (Fig. 3C) [18]. This evidence pointed out to chronic TDP-43 LLPS – not only aggregation – as a source on neurotoxicity, even without a direct transition to aggregated species. Consistently, Bolognesi and collaborators observed the formation of toxic liquid-like TDP-43 condensates in the cytoplasm of yeast cells, clustered at the nuclear periphery, upon introducing an array of mutations in the PrLD of full-length TDP-43 [152].

Interestingly, TDP-43 is also present in cytoplasmic, non-phase-separated, SDS-resistant amyloid-like ribonucleoprotein assemblies known as myo-granules, considerably different from MLOs, occurring in regenerating muscle fibres [153]. These ordered structures, enriched in RNA-binding proteins and mRNAs encoding sarcomeric structural proteins to which TDP-43 preferentially binds, play a cardinal role in skeletal muscle functionality. Unlike pathological aggregates, which persist in cells, myo-granules are physiologically cleared as myofibers are repaired [153].

### Paraspeckles, where TDP-43 acts as a molecular switch for their level of assembly

Paraspeckles are protein-rich nuclear bodies built around an architectural long noncoding RNA scaffold known as nuclear paraspeckle assembly transcript 1 (NEAT1) (Fig. 4A) [154, 155]. They are involved in the regulation of gene expression by sequestering specific RNAs and proteins and in miRNA biogenesis [156]. Among NEAT1 protein interactors, an array of ALS-linked proteins, including TDP-43, has been described [21, 155, 156]. Paraspeckles accumulate upon stress exposure and in motoneurons of ALS subjects [157–159]. Specifically, TDP-43 disperses paraspeckles when it is in excess in an RNA-binding dependent manner, whereas in stressed cells the protein is recruited to de novo stress-induced nuclear condensates and paraspeckles are upregulated, as the protein has an inhibitory effect [160]. An increased proportion of TDP-43 containing paraspeckles can also form under conditions in which TDP-43 is not excessive [160] by the RNA-nucleated TDP-43 self-assembly on UG repeats in the NEAT1_2 isoform of NEAT1, which enables TDP-43 partitioning in micro-condensates with FUS [21, 161]. In this case, paraspeckles are less dynamic and tend to form clusters [161]. Both paraspeckle suppression and/or formation of excessive TDP-43-containing paraspeckles were reported to attenuate their response to stress conditions, thus promoting ALS progression [160].

### Nuclear stress bodies and other TDP-43 stress-induced nuclear condensates

Cellular stress response involves a profound reorganization of the nuclear structure, characterised by the formation of de novo condensates such as NSBs [162] (Fig. 4A). NSBs positive for the heat shock factor 1 (HSF1) and the scaffold attachment factor B (SAFB) are densely packed RBP organelles forming at DNA loci containing non-coding satellite III tandem repeat sequences and at pericentromeric sites in response to cellular stress [163]. Their liquid nature has been shown by their disassembly upon 1,6 hexanediol [164] and both 1,6- and 2,5-hexanediol treatment [165] and high relative fluorescence recovery in FRAP experiments [164, 165]. TDP-43 was found to be recruited into NSBs through reversible C-terminal-mediated aggregation under acute stress, leading to its functional, reversible inactivation associated with a transient loss of binding to its protein interactors and function in RNA processing [24]. More recently, TDP-43 containing nuclear condensates, devoid of RNA and liquid properties, have been described to form using sodium arsenite as a trigger, but they do not colocalize with NSBs or other known constitutive NBs, despite being located in their proximity and physically interacting with them, suggesting a possible TDP-43 transfer among these structures [166].

### Anisosomes as disease-associated RNA-void droplets maintained by ATP-dependent Hsp70

Very recently, an array of human cell lines was used to demonstrate that TDP-43, either mutated or characterised by an increased acetylation with consequent deficient RNA-binding-capability, undergoes a distinct type of LLPS, demixing into liquid anisotropic spherical shells in the nucleus (Fig. 4A) [25]. Such RNA-void droplets, referred to as anisosomes, are enriched in TDP-43 in the outer shell and contain HSP70 chaperones in the interior [25]. The structure and fluidity of anisosomes is guaranteed by the adenosine triphosphatase (ATPase)–dependent chaperone activity of Hsp70 chaperones. A loss of such activity, occurring with ageing or cellular stressors, may cause the solidification of anisosomes, seed TDP-43 aggregation and thus they may act as precursors of nuclear and/or cytoplasmic TDP-43 aggregates found in ALS and other TDP-43-associated proteinopathies [25] (Fig. 4B). TDP-43 anisosomes are paradigmatic, as they are very similar to RNA-containing anisotropic nuclear condensates formed as aggregation intermediates of the poly-PR peptide derived from ALS/FTD-linked C9orf72 gene mutations, that were also reported to trigger abnormal nuclear granulation of TDP-43 [167].

## Conclusions

In the last decade, tremendous efforts have been made to investigate the role of both functional and dysfunctional LLPS and their pathological consequences in neurodegeneration. Among the numerous proteins undergoing LLPS, TDP-43 is one of the most extensively characterised, thus serving as a model protein to study phase separation dynamics both in vitro and in vivo. Unlike PrLD and PrLD-less fragments, pure full-length TDP-43 does not form stable liquid droplets, with their stable formation requiring RNAs, chaperones or possibly other co-factors. Electrostatic and hydrophobic forces determine TDP-43 phase separation, similarly to other protein systems. The ability of TDP-43 to undergo LLPS into both cytoplasmic and nuclear condensates, with distinct structural and biological properties, directly allows the modulation of protein functionality. However, in pathogenic conditions, aberrant phase transitions might lead to the formation of both liquid and solid neurotoxic entities. Although these issues are still debated and substantial evidence exists that both physiological and stress-induced MLOs have neutral or beneficial functional effects, evidence is mounting that solid inclusions can form from such liquid MLOs.

## Data Availability

No datasets were generated or analysed during the current study.
